# Collective cell migration of smooth muscle and endothelial cells: impact of injury versus non-injury stimuli

**DOI:** 10.1186/s13036-015-0015-y

**Published:** 2015-10-15

**Authors:** Kaitlyn R. Ammann, Katrina J. DeCook, Phat L. Tran, Valerie M. Merkle, Pak K. Wong, Marvin J. Slepian

**Affiliations:** Biomedical Engineering GIDP, University of Arizona, Tucson, AZ USA; Sarver Heart Center, College of Medicine, University of Arizona, 1501 N Campbell Ave, Tucson, AZ 85724 USA; Department of Aerospace and Mechanical Engineering, University of Arizona, Tucson, AZ USA; Department of Biomedical Engineering, University of Arizona, Tucson, AZ USA

**Keywords:** Collective cell migration, Injury, Vascular, Smooth muscle cell, Endothelial cell, Scrape wound, Gelatin, Polystyrene

## Abstract

**Background:**

Cell migration is a vital process for growth and repair. *In vitro* migration assays, utilized to study cell migration, often rely on physical scraping of a cell monolayer to induce cell migration. The physical act of scrape injury results in numerous factors stimulating cell migration – some injury-related, some solely due to gap creation and loss of contact inhibition. Eliminating the effects of cell injury would be useful to examine the relative contribution of injury versus other mechanisms to cell migration. Cell exclusion assays can tease out the effects of injury and have become a new avenue for migration studies. Here, we developed two simple non-injury techniques for cell exclusion: 1) a Pyrex® cylinder - for outward migration of cells and 2) a polydimethylsiloxane (PDMS) insert - for inward migration of cells. Utilizing these assays smooth muscle cells (SMCs) and human umbilical vein endothelial cells (HUVECs) migratory behavior was studied on both polystyrene and gelatin-coated surfaces.

**Results:**

Differences in migratory behavior could be detected for both smooth muscle cells (SMCs) and endothelial cells (ECs) when utilizing injury versus non-injury assays. SMCs migrated faster than HUVECs when stimulated by injury in the scrape wound assay, with rates of 1.26 % per hour and 1.59 % per hour on polystyrene and gelatin surfaces, respectively. The fastest overall migration took place with HUVECs on a gelatin-coated surface, with the in-growth assay, at a rate of 2.05 % per hour. The slowest migration occurred with the same conditions but on a polystyrene surface at a rate of 0.33 % per hour.

**Conclusion:**

For SMCs, injury is a dominating factor in migration when compared to the two cell exclusion assays, regardless of the surface tested: polystyrene or gelatin. In contrast, the migrating surface, namely gelatin, was a dominating factor for HUVEC migration, providing an increase in cell migration over the polystyrene surface. Overall, the cell exclusion assays - the in-growth and out-growth assays, provide a means to determine pure migratory behavior of cells in comparison to migration confounded by cell wounding and injury.

**Electronic supplementary material:**

The online version of this article (doi:10.1186/s13036-015-0015-y) contains supplementary material, which is available to authorized users.

## Background

Cell migration plays a vital, fundamental role in growth, differentiation and repair of normal and diseased tissues. It is a complex, cyclical process that is dependent upon the delicate balance of multiple mechanisms [1–3]. In particular, the degree of injury stimulation, paracrine growth factor and mediator release, substrate surface properties, or the loss of contact inhibition, all sway the balance to either inhibit or enhance migration [3, 4]. These mechanisms are of particular relevance to group migration of cells on a substrate, what is termed collective cell migration [5]. Understanding and manipulation of collective cell migration and its contributory mechanisms offers utility as a means of modulating pathophysiological processes such as wound healing.

The two-dimensional, scrape wound assay is the traditional technique utilized to study collective flat sheet migration [6]. The act of scraping the cell monolayer imparts a physical injury stimulus to the monolayer, releasing cellular contents into the cell media [7, 8]. Gap formation by injury results in several processes that drive migration including: the loss of contact inhibition, creation of a free edge for directional migration, disruption of the matrix and the release of local cell debris, i.e. membranes and stored growth factor and mediator [6, 8–10]. Cells on the leading edge of the newly created gap typically respond to these biochemical and physical signals by migrating directionally into the wound to close the gap, establishing new cell-cell contacts and regenerating a cell monolayer [6, 9, 10]. Although the scrape wound assay is a quick and easy technique to examine migration, measurement of migration with this assay is the net sum of all operating mechanisms outlined above. As such, it is limited by the inability to control the multiplicity of concomitant variables, mechanisms and effects ongoing simultaneously.

To limit and control the impact of component mechanisms and factors on the migratory process, additional assays are needed which afford control of these variables, stimulating migration via fewer mechanisms. Cell exclusion migration assays have become a popular, simple and inexpensive means of studying migration [11–19]. These assays involve release of contact inhibition for an adjacent cell monolayer via removal of an anti-migratory gate or dam to initiate migration. However, only few of these studies have directly compared the collective migratory differences between non-injury and traditional injury models [12, 13, 15]. While an in-growth type assay and out-growth type assay have been compared to injury models separately, there lacks a comprehensive work in which multiple assays are utilized [12, 13, 15]. This is an important aspect due to the high variability of results present among such assays, especially with the scrape wound [7, 8].

Furthermore, there lacks a focus in the current literature on such a comparison between endothelial and smooth muscle cell types: two cells that are functionally inter-dependent and commonly involved in wound healing-associated diseases [20]. There is a strong consensus that vascular SMCs switch to a proliferative phenotype when stimulated by physical force or injury that leads to an increase in proliferative and migratory rates [20–22]. However, the injury effects on ECs have been shown to be much more variable, and are sensitive to substratum differences and endothelial dysfunction [22–24]. In a study done by Van Horssen, et al. collective EC migration increased when stimulated by injury versus non-injury [12]. In contrast, Hulkower, et al. described a decrease in migration post-injury compared to non-injury [7]. In an effort to further elucidate the migration of vascular cells, a direct comparison needs to be explored in which non-injury and injury effects are examined across multiple assays and substrata for both vascular cell types.

In the present study, we describe two simple types of non-injury migration assays that employ either cell exclusion or anti-migratory gates, which do not rely on injury as an initiating stimulus. Herein, utilizing these two assays, we compare collective cell migration of vascular cells, i.e. smooth muscle cells (SMCs) and human umbilical vein endothelial cells (HUVECs), with that observed with the conventional scrape injury. Additionally, we examined the effect of substrate on migration in each of these assays. Utilization of each of these assays affords the possibility of teasing out the relative contribution of several contributory factors to the migratory process of vascular cells.

## Results

### Non-injury assay fabrication and validation

**Fig. 1:**
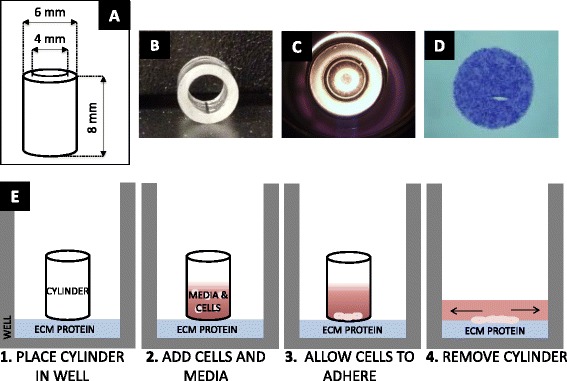
Out-growth assay. **a** Layout of Pyrex® cylinder with height and inner diameter dimensions. **b** Photo of Pyrex® cylinder. **c** Bottom view of cylinder inside well with cells seeded inside. **d** Photo of stained cells 0 h after removal of cylinder. **e** Sequence of steps to perform out-growth assay (Steps 1–4)

**Fig. 2:**
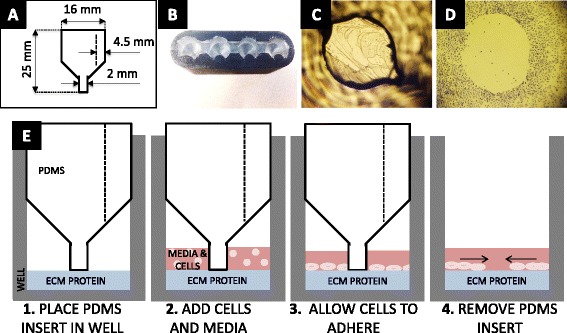
In-growth assay. **a** Layout of PDMS insert with lower and upper diameter dimensions. **b** Top view photo of PDMS insert inside of mold. **c** Bottom view photo of PDMS insert inside a well with cells seeded around. **d** Image of stained cells 0 h after PDMS insert removal. **e** Sequence of steps to perform in-growth assay (Steps 1–4)

Two convenient, reproducible non-injury assays were developed, allowing use of conventional 24-well plates as a cell growth substrate, combined with readily available lab materials, e.g. Pyrex® cylinders and PDMS forms. One assay involved containment of cells in a defined space, which upon removal allowed outward migration – termed the out-growth assay (Fig. 1). The second method involved creating an exclusion zone in a dish during cell seeding, via placement of a deformable PDMS form, which upon removal created a cell-free zone – termed the in-growth assay (Fig. 2). For the out-growth assay (*n* > 100 use cases), cell seeding within the cylinder was not associated with leakage of inoculum. Upon removal of the cylinder in all cases, a discrete cell zone leading edge was created and readily identified (Fig. 1d), allowing quantitative measurement and tracking of out-migration at subsequent incubation time points. Similarly for the in-growth assay, all cases of PDMS form removal following cell seeding led to a clearly detectable cell-free circular zone with a clean and defined migrating edge (Fig. 2d). This too allowed ready measurement of subsequent in-migration relative to the original border.

### Collective cell migration – Non-injury versus injury

**Fig. 3:**
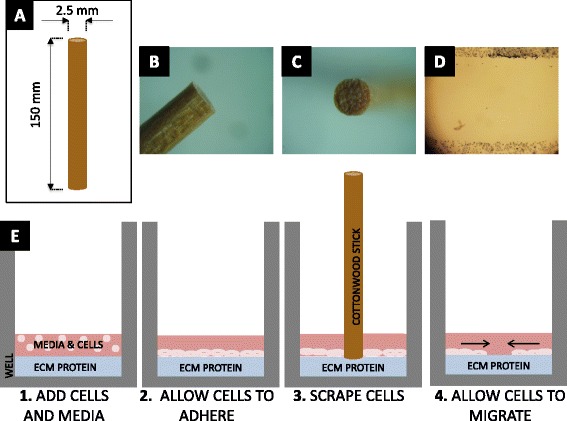
Scrape wound assay. **a** Layout of cottonwood stick with height and diameter dimensions. **b** Side view photo of cottonwood stick tip. **c** Top view of cottonwood stick tip. **d** Image of stained cells 0 h after scratch. **e** Sequence of steps to perform scrape wound assay (Steps 1–4)

**Fig. 4:**
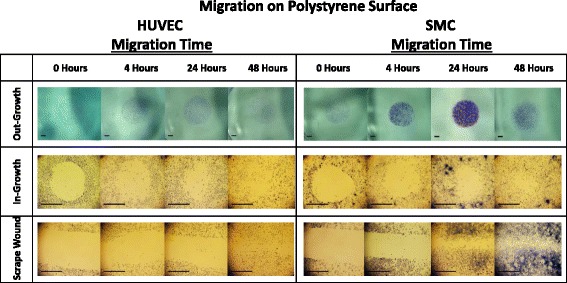
Image table of migrating HUVEC and SMC. Images depict increasing cell area as cells migrate after 0, 4, 24, and 48 h (*left* to *right*). Migration is shown on polystyrene surface for the out-growth assay (*top row*), in-growth assay (*middle row*), and scrape wound assay (*bottom row*). Black scale bar (*lower left*) in each image represents 1 mm

**Fig. 5:**
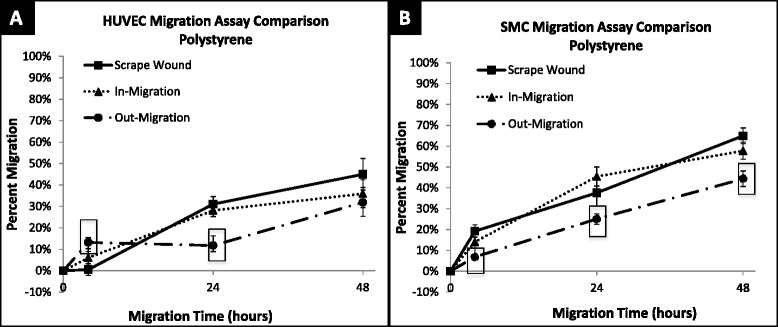
Percent migration of HUVEC and SMC across 48 h on polystyrene. **a** HUVEC migration on polystyrene surface at 4, 24 and 48 h; shown in percent migration when compared to baseline migration at 0 h. Out-migration and in-migration were significantly higher than scrape wound migration at 4 h, (*p* < 0.0001 and *p* = 0.0183). Out-migration was significantly lower than scrape wound migration at 24 h (*p* = 0.0014). **b** SMC migration on polystyrene surface at 4, 24, and 48 h; shown in percent migration when compared to baseline migration at 0 h. SMC out-migration was significantly lower than scrape wound migration at 4 (*p* = 0.0020), 24 (*p* = 0.0464) and 48 (*p* = 0.0003) hours. ☐ symbol outline indicates statistical significance (*p*-values < 0.05). Values shown as mean ± standard error

Smooth muscle and endothelial cell migration were examined utilizing the described non-injury assays (Figs. 1 and 2) compared to scrape wound (Fig. 3). Following initiation of migration, i.e. cylinder or PDMS insert form removal, the regions of new cell out-growth or in-growth (cell-occupied areas) at 4, 24, and 48 h of migration were measured. Representative images of the migrated cells on polystyrene are shown in Fig. 4. Using these area measurements, the percent migration was calculated and averaged for each assay and time point for both HUVECs and SMCs, (Fig. 5a and b). Despite the variability in cell seeding area, all three assays were seeded at a constant concentration prior to initiation of migration. This led to a variation in cell density among the assays that proved to have insignificant effects on the migration of the cells (Additional file 1).

### HUVECs

**Fig. 6:**
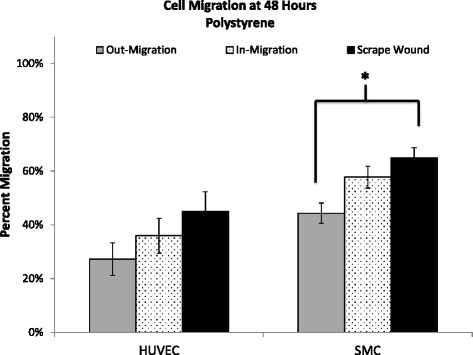
Percent migration of HUVEC and SMC at 48 h on polystyrene. Percent migration is calculated by comparison to baseline migration at 0 h. Out-migration and scrape sound migration of SMCs at 48 h are significantly different (*p* = 0.0003). Values shown as mean ± standard error

After 4 h of migration, both non-injury assays had significantly higher migration than the injury scrape wound assay with HUVECs (6.02 ± 4.24 %, *p* = 0.018 in-growth assay and 13.21 ± 2.28 %, *p* < 0.001 out-growth assay, Fig. 5a). However, after 48 h, the percent migration of HUVECs was not significantly different between the two non-injury assays and the injury assay (out-growth: 31.91 ± 6.92 % vs. scrape wound: 45.02 ± 7.30 %, *p* = 0.110; in-growth: 35.95 ± 6.51 % vs. scrape wound: 45.02 ± 7.30 %, *p* = 0.188) (Fig. 6).

### SMCs

SMCs showed more consistent differences across migration assay type and over time than that observed with HUVEC. The non-injury out-growth assay exhibited significantly lower migration compared to the injury scrape wound assay at 4 h (out-growth: 6.86 ± 0.99 % vs. scrape wound: 19.25 ± 2.93 %, *p* = 0.0008), 24 h (out-growth: 25.05 ± 2.54 % vs. scrape wound: 37.60 ± 6.28 %, *p* = 0.046), and 48 h (out-growth: 44.33 ± 3.76 % vs. scrape wound: 64.96 ± 3.76 %, *p* = 0.0003) (Fig. 5b). In contrast, there was no significant difference in SMC migration observed between non-injury in-growth and scrape wound for the time points studied.

Regardless of these differences, the trend at 48 h for both HUVECs and SMCs was the same, i.e. the scrape wound assay – i.e. the injury assay, provided the highest percent migration at 48 h (HUVEC: 45.02 ± 7.30 %, SMC: 64.96 ± 3.76 %) for both cell types. In contrast the non-injury assays resulted in lower levels of migration, with the in-growth assay affording greater migration (HUVEC: 35.95 ± 6.51 %, SMC: 57.78 ± 4.07 %) than the out-growth assay (HUVEC: 31.91 ± 6.92 %, SMC: 44.33 ± 3.76 %) for both cell types (Fig. 6).

### Effect of substrate on Non-injury versus injury-mediated migration

**Fig. 7:**
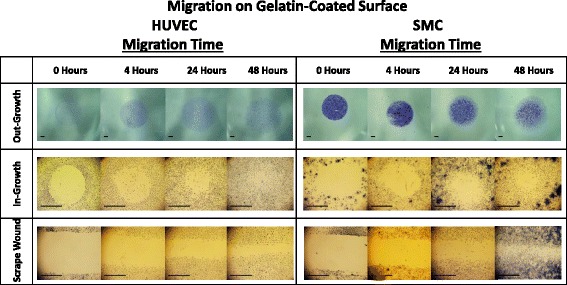
Image table of migrating HUVEC and SMC. Images depict increasing cell area as cells migrate after 0, 4, 24, and 48 h (*left* to *right*). Migration is shown on a gelatin surface for the out-growth assay (*top row*), in-growth assay (*middle row*) and scrape wound assay (*bottom row*). Black scale bar (*lower left*) in each image represents 1 mm

**Fig. 8:**
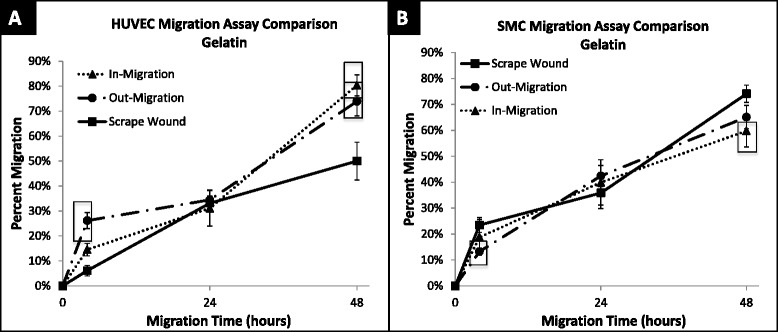
Percent migration of HUVEC and SMC across 48 h. **a** HUVEC migration on gelatin surface at 4, 24 and 48 h; shown in percent migration when compared to baseline migration at 0 h. In-migration was significantly larger than scrape wound migration at 4 (*p* = 0.0075) and 48 (*p* = 0.0014) hours. Out-migration was significantly larger than scrape-wound migration at 4 (*p* < 0.0001) and 48 (*p* = 0.0106) hours. **b** SMC migration on gelatin surface at 4, 24, and 48 h; shown in percent migration when compared to baseline migration at 0 h. Out-migration was significantly lower than migration of scrape wound at 4 h (*p* = 0.0026). In-migration was significantly lower than scrape wound migration at 48 h (*p* = 0.0275). ☐ symbol outline indicates statistical significance (*p*-values < 0.05). Values shown as mean ± standard error

**Fig. 9:**
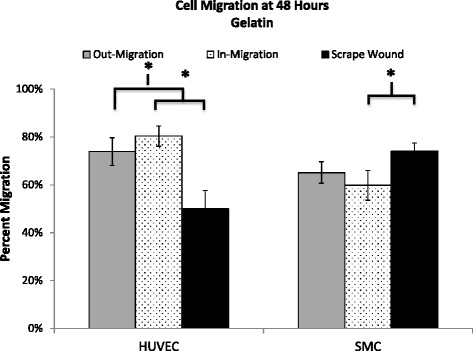
Percent migration of HUVEC and SMC at 48 h on gelatin. Percent migration is calculated by comparison to baseline migration at 0 h. HUVECs showed significant difference between scrape wound and out-migration (*p* = 0.0106), and scrape wound and in-migration (*p* = 0.0014). SMCs showed significant difference between scrape wound and out-migration (*p* = 0.0275). Values shown as mean ± standard error

Non-injury versus injury-mediated migration of HUVECs and SMCs on a gelatin-coated surface can be seen qualitatively in Fig. 7 and quantitatively in Fig. 8a and b. HUVEC migration on a gelatin-coated surface, at 48 h revealed the following percent migration: 73.90 ± 5.81 % for the out-growth assay, 80.36 ± 4.20 % for the in-growth assay, and 49.99 ± 7.62 % for the scrape wound assay (Fig. 9a). In contrast, SMCs demonstrated 65.12 ± 4.48 % outward migration, 59.78 ± 6.21 % inward migration, and 74.08 ± 3.37 % inward migration with the injury scrape wound assay (Fig. 9b).

**Fig. 10:**
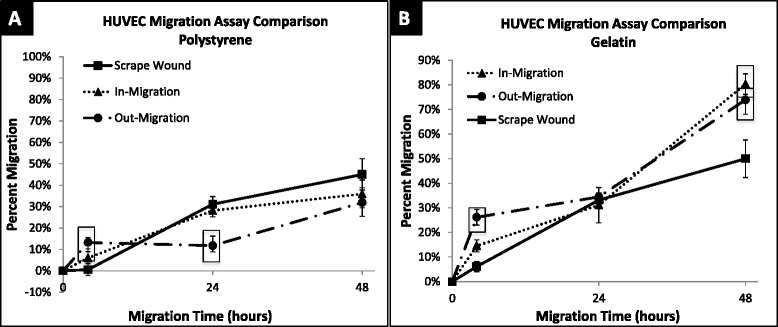
Percent migration of HUVEC across 48 h on polystyrene and gelatin. **a** HUVEC migration on polystyrene surface at 4, 24 and 48 h; shown in percent migration when compared to baseline migration at 0 h. Out-migration and in-migration were significantly higher than scrape wound migration at 4 h, (*p* < 0.0001 and *p* = 0.0183). Out-migration was significantly lower than scrape wound migration at 24 h (*p* = 0.0014). **b** HUVEC migration on gelatin surface at 4, 24 and 48 h; shown in percent migration when compared to baseline migration at 0 h. In-migration significantly larger than scrape wound migration at 0 (*p* = 0.0075) and 48 (*p* = 0.0014) hours. Out-migration significantly larger than scrape wound migration at 0 (*p* < 0.0001) and 48 (*p* = 0.0106) hours. ☐ symbol outline indicates statistical significance (*p*-values < 0.05). Values shown as mean ± standard error

**Fig. 11:**
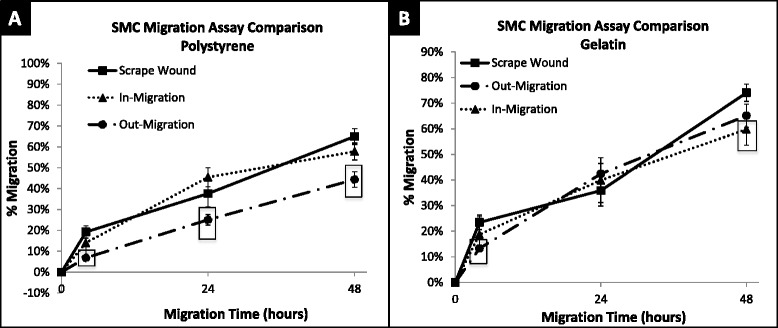
Percent migration of SMC across 48 h on polystyrene and gelatin. **a** SMC migration on polystyrene surface at 4, 24, and 48 h; shown in percent migration when compared to baseline migration at 0 h. SMC out-migration was significantly lower than scrape wound migration at 4 (*p* = 0.0020), 24 (*p* = 0.0464) and 48 (*p* = 0.0003) hours. **b** SMC migration on gelatin surface at 4, 24, and 48 h; shown in percent migration when compared to baseline migration at 0 h. Out-migration was significantly lower than migration of scrape wound at 4 h (*p* = 0.0026). In-migration was significantly lower than scrape wound migration at 48 h (*p* = 0.0275). ☐ symbol outline indicates statistical significance (*p*-values < 0.05). Values shown as mean ± standard error

The gelatin substrate had a clear impact in modulating migration. HUVECs on the gelatin-coated surface showed a significant increase in percent migration compared to polystyrene, with a change from 32 % to 44 % (42 % increase), a change from 36 to 80 % (44 % increase), and a change from 45 to 50 % (5 % increase) observed for the out-growth, in-growth, and scrape wound assays, respectively (Figs. 8 and 10). HUVEC migration with the scrape wound assay was not as significantly affected by the gelatin substrate at 48 h compared to the two non-injury assays, causing them to be significantly different, with *p* = 0.016 for the out-growth assay and *p* = 0.0014 for in-growth assay (Fig. 10a). In contrast, the underlying gelatin substrate did not induce as significant an increase in migration for SMCs at 48 h, with an increase of only 21, 2, and 9 % for the out-growth, in-growth and scrape wound assays, respectively (Fig. 11). Regardless, it is clear that cells preferred to migrate across the gelatin-coated substrate (Fig. 7), migrating further than when seeded on polystyrene (Fig. 4).

Gelatin also led to enhanced migratory rates, as can be seen especially with HUVECs. The migratory rates of cells on gelatin-coated surfaces between 24 and 48 h are given in Table 1. SMC migration with the scrape wound assay had a 1.59 % per hour migratory rate on gelatin, while HUVECs with the scrape wound assay had the lowest migratory rate on the gelatin-coated surfaces, with a rate of only 0.70 % per hour (Table 1). Interestingly, HUVECs on gelatin in the case of the in-growth assay exhibited the highest migration rate across all surfaces, cell types, and assays with a migratory rate of 2.05 % per hour, and the lowest with the same assay when on polystyrene at a rate of 0.33 % per hour (Tables 1 and 2).

**Table 1:** Average rate of percent cell migration on gelatin from 24–48 h

Assay	HUVEC (% migration/hr)	SMC (% migration/hr)
Out-Growth	1.29 ± 0.05	0.95 ± 0.04
In-Growth	2.05 ± 0.06	0.83 ± 0.07
Scrape Wound	0.70 ± 0.05	1.59 ± 0.05

Rates multiplied by 100 as a display of simplicity. HUVEC had highest migration rate with in-growth assay (2.05 %/hour), SMC highest migration rate with scrape wound assay (1.59 %/hour). Values shown as average ± standard error

**Table 2:** Average rate of percent cell migration on polystyrene from 24–48 h

Assay	HUVEC (% migration/hr)	SMC (% migration/hr)
Out-Growth	0.72 ± 0.06	0.80 ± 0.03
In-Growth	0.33 ± 0.05	0.51 ± 0.04
Scrape Wound	0.58 ± 0.05	1.26 ± 0.05

Rates multiplied by 100 as a display of simplicity. HUVECs had highest migration rate with out-growth assay (0.72 %/hour). SMCs highest migration rate with scrape wound assay (1.26 %/hour). Values shown as average ± standard error

**Fig. 12:**
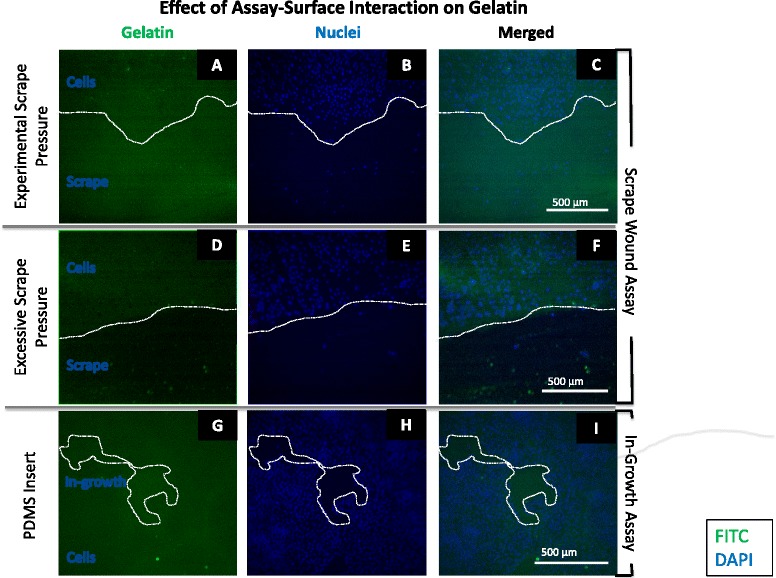
Image table of SMC migration on gelatin-FITC after 24 h. **a**-**c** Images depict wound edge of scrape wound assay at experimental scrape pressure on gelatin-FITC-coated surface. **d**-**f** Images depict wound edge of scrape wound assay at excessive scrape pressure on gelatin-FITC-coated surface. **g**-**i** Images depict cell in-growth area at experimental PDMS insert pressure on gelatin-FITC-coated surface. White dashed line outlines current wound edge. White scale bar (lower right in each row) represents 500 μm. Gelatin (green); SMC Nuclei (blue)

To ensure that migratory results were not affected by forces exerted on the gelatin-coated surface during the scrape wound and in-growth assays, gelatin-FITC substratum was examined after a 24 h migratory time point for these two assays (Fig. 12). The gelatin remained adherent to the surface after being scraped (Fig. 12a-c) and coming in contact with the PDMS insert (Fig. 12h-i) at experimental pressures (7 g/mm^2^ or less). This is denoted by the confluent green fluorescence post scratch (Fig. 12a) and post lift-off of the PDMS insert (Fig. 12g). However, when excessive pressure was applied (190 g/mm^2^ or greater), scraping of the surface removed gelatin (Fig. 12d-f).

### HUVEC and SMC comparison

The effects of cell type on percent migration and migratory rate were also examined in our comparative analysis. The migratory rates of SMCs and HUVECs on polystyrene between 24 and 48 h are shown in Table 2. SMCs were observed to have the fastest migration rates after being subjected to injury in the scrape wound assay, with a 1.26 % per hour migratory rate (Table 2). In contrast, HUVECs did not have a positive injury response and showed a much lower migratory rate than SMCs with the scrape wound assay, migrating at 0.58 % per hour on polystyrene (Table 2). These results were similar when comparing across non-injury assays, as HUVECs continued to show a lower migration rate when compared to SMCs with 0.72 % per hour for the out-growth assay and 0.33 % per hour for the in-growth assay (Table 2). This trend was consistent when comparing overall percent migration, as SMCs had higher overall migration after 48 h for the in-growth assay (57.78 ± 4.07 %), the out-growth assay (44.33 ± 3.76 %), and the scrape wound assay (64.96 ± 3.76 %), in comparison to HUVEC migration on polystyrene after 48 h (Fig. 6).

Of both cell types and substrates evaluated, HUVECs had the highest migration on the gelatin surface but the lowest on polystyrene when studied in the non-injury migration assays (Table 3). However, when injury was taken into account with the scrape wound assay, SMCs had the highest migration on the gelatin surface while HUVECs had the lowest amount of migration on polystyrene (Table 3).

**Table 3:** Summary of description of each assay and corresponding results

Assay	Injury?	Wound shape	Initial wound size	Highest migration	Lowest migration
Scrape Wound	Yes	Rectangle	d = 2.5 mm	SMC on gelatin	HUVEC on polystyrene
Out-Growth	No	Circle	d = 4 mm	HUVEC on gelatin	HUVEC on polystyrene
In-Growth	No	Circle	d = 2 mm	HUVEC on gelatin	HUVEC on polystyrene

Description categories include whether the assay creates injury, what shape of wound it creates, and the size of the wound. Best overall migration rates after 48 h are also displayed according to cell type and surface substrate

**Fig. 13:**
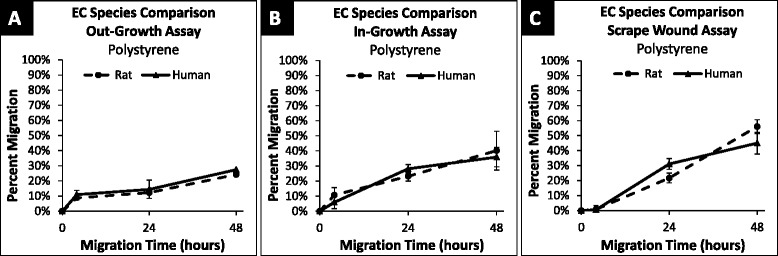
Percent migration of RVEC and HUVEC across 48 h on polystyrene. **a** RVEC and HUVEC migration on polystyrene surface using the out-growth assay at 4, 24 and 48 h; shown in percent migration when compared to baseline migration at 0 h. No significant difference between species at 4 h (*p* = 0.13), 24 h (*p* = 0.14), or 48 h (*p* = 0.29). **b** RVEC and HUVEC migration on polystyrene surface using the in-growth assay at 4, 24 and 48 h; shown in percent migration when compared to baseline migration at 0 h. No significant difference between species at 4 h (*p* = 0.48), 24 h (*p* = 0.26), or 48 h (*p* = 0.78). **c** RVEC and HUVEC migration on polystyrene surface using the scrape wound assay at 4, 24 and 48 h; shown in percent migration when compared to baseline migration at 0 h. No significant difference between species at 4 h (*p* = 0.06), 24 h (*p* = 0.06), or 48 h (*p* = 0.18). Values shown as mean ± standard error

To further elucidate the comparison of injury and non-injury factors on smooth muscle and endothelial cell types, the assays were repeated using rat vein endothelial cells (RVECs) (Fig. 13). These results showed no significant difference in migration between the HUVECs and RVECs with any of the three assays at any time point. As such, it can be concluded that there is no distinguishable effect of rat or human species on the migratory behavior in these cases.

## Discussion

Utilizing easily constructed non-injury assay methods, differences in cell behavior were revealed between non-injury versus injury-mediated initiation of collective cell migration. A goal of this study was to develop a simple, reproducible means to isolate and control aspects of the initiating and driving stimuli for migration of cell sheets on a 2-D surface. Our data supports the efficacy of creating containment or exclusion zones of cells in a culture preparation as a means of creating a leading edge from which to quantitatively track collective cell movement. Further, removal of inserts provides a means of physically initiating cell migration free of conventional scrape wound injury effects. With this system, we were able to differentiate the contribution of factors including: injury, substrate, and cell type on collective cell migration. We observed that injury led to a positive migratory response for SMCs, but a less significant response for HUVECs. For SMCs, it appears that injury is a dominating factor in driving migration when compared to the two non-injury assays, regardless of the substrate tested. In contrast, the underlying substrate, namely gelatin, was a dominating factor for HUVEC migration, providing an increase in cell migration in comparison to that observed on the polystyrene surface.

### Collective cell migration – Non-injury versus injury methods

Previously, various methods such as oil drop assays, mesh ring assays, and barrier exclusion assays were utilized to quantify migration and study non-injury physiological processes such as embryonic development, angiogenesis, and cancer metastasis [11, 13–19]. Riahi et. al, in reviewing these methods reported that the cell exclusion approach typically provides reproducible assays that can be standardized, minimizing cellular injury in comparison to the scrape wound assay [8]. Our observations concur with this perspective. However, our approach goes beyond that reported previously in that the simple assay methods in this study create two opposing condition of cells collectively migrating, i.e. 1) away from a cell source, i.e. out-growth assay, or 2) inwards from a source, i.e. in-growth assay. Further, while not specifically experimentally tested here as to biochemical or mediator mechanisms, these assay constructs create conditions of cells either moving away from a concentration gradient of local paracrine or other contiguous mediator effects (out-growth assay) or conversely conditions of cells moving inward, towards an increasing concentration gradient of paracrine or mediator effects (in-growth assay).

In the scrape wound assay, injury to a confluent layer of cells, dislodges and variably ruptures cells, causing them to release intracellular content [12, 16]. Sudden influx of intracellular contents and release of mediators can trigger migration stimulating a wound healing process [16]. Injury in this assay is typically variable, being influenced by the degree of pressure exerted and the extent of manual control of the scraping of the monolayer. The scraping tool utilized as well as the velocity of the scrape all affect the amount of cellular damage, consequently affecting migratory behavior [7, 25]. These variable effects of injury often necessitate multiple replicate experiments to provide a clearly observed effect. In our study all experiments were done with an *n* = 20 per time point to yield a clear effect signal. In contrast, in the non-injury assays, with a clear sharp cell leading edge established, clearer migratory effects are observed with a lower number of replicate experiments.

### Effect of substrate on Non-injury versus injury-mediated migration

As cell migration is dependent upon a balance between adhesion and release from the substrate surface, surface characteristics and composition are additional key factors that affect migratory behavior. We compared migration on tissue culture polystyrene with that on gelatin, under both injury and non-injury migration conditions to elucidate the contribution of a biologically active surface to injury and non-injury migration.

The polystyrene surface in this study was plasma-treated with a Nunclon® delta surface treatment. This surface treatment changes the hydrophobic and unfavorable surface to a more hydrophilic surface on which cells can adhere [25]. In contrast, biological substrata such as gelatin provide a surface with a higher wettability and availability of adhesion sites [4]. Cells gain traction for migration on gelatin from integrin-based focal adhesions at RGD attachment sites present on the gelatin structure [25–28]. While protein adsorption to the polystyrene surface can allow for binding to cell integrins, it is not as favorable or as strong, as the integrin binding of the gelatin-coated surface [27].

Our results clearly support the favorable effects of cell-protein, i.e. largely integrin-matrix interactions, for HUVECs, with a significant increase in migration observed at 48 h on gelatin compared to polystyrene. Similarly, the rate at which the HUVECs migrated increased across all assays in moving to a gelatin surface (Table 1) compared to a polystyrene (Table 1) surface. In contrast, the contribution of an underlying protein (gelatin) surface, with attendant integrin-matrix interactions, was less significant as a major modulator of observed migration for SMCs. (Figs. 8 and 11). Although the present study examines the integrin-matrix interactions between vascular cells and gelatin specifically, our assays can easily be extended to examine a variety of ECM proteins. Our future work includes examination of a variety of ECM proteins relevant to the vascular wall.

### HUVEC and SMC comparison

From the differing responses of SMC and HUVEC to injury, it is clear that injury and substrate affect these cell types differently; SMCs had a stronger reaction to injury than HUVECs, while HUVECs had a stronger response to substrata than SMCs.

Endothelial cells are heavily anchorage-dependent, utilizing integrin-matrix interactions for stability [24]. Therefore, when a substrate such as gelatin is provided for HUVECs, under pro-migratory conditions an increase in migration is expected [29–31]. Our results are consistent with this, with HUVECs demonstrating an increase of percent migration on gelatin compared to polystyrene for all assays. In contrast, the increase observed with the scrape wound assay was not nearly as significant as the difference seen in the non-injury assays (out-growth and in-growth), revealing that the gelatin-coated surface rather than injury had a more significant effect, as a driving mechanism, on the migration rate of HUVECs. These observations suggest that HUVECs are more sensitive and responsive to the surface modification tested, i.e. gelatin coating, than SMCs and exhibit a substantial preference for a gelatin surface over polystyrene [29–31].

With respect to endothelial cells, injury can also result in growth-stimulatory characteristics but also changes to permeability and adhesive characteristics which can hinder their ability to migrate [4, 22]. The injury response of HUVECs on polystyrene support a conclusion in which injury caused a hindered ability to migrate for HUVECs but a more robust rate for SMCs during the late stages of migration (24 and 48 h).

The robust response of SMCs to injury is supported in the literature, as they have been shown to respond to bioactive molecules from injury, causing them to migrate and proliferate at alarming rates in vessels [22]. Bioactive molecules that can be released into the medium post-injury include growth factors, e.g. platelet-derived growth factor (PDGF), basic fibroblast growth factor (bFGF), epidermal growth factor (EGF), transforming growth factor ẞ-1 (TGF-ẞ), and insulin-like growth factor (IGF-1), which all serve as chemoattractants for migration [32]. These are factors sourced by both ECs and SMCs which upon rupture can cause an immediate spike in concentration of these factors in surrounding media. This in turn leads to an expected migration stimuli [21, 22]. While this is a potent mediator for SMCs, another growth factor—vascular endothelial growth factor (VEGF) is the potent regulator for ECs. This factor, however, is released from platelets that are not present in the cell media and may explain the less robust response we see in the results [22–24, 33]. Injury effects from SMCs can also include ECM protein deposition and phenotypic changes that lead to enhanced migration [26]. Cells at the injury edge can undergo morphological changes, becoming rough, non-uniform, forming membrane extensions that lead to motility [34].

### Clinical implications

Our translational interest in injury-stimulated cell migration stems from limitations remaining in the treatment of atherosclerosis and post-intervention restenosis. Atherosclerosis, a leading cause of mortality and morbidity worldwide is commonly treated by balloon angioplasty or stent deployment in arteries, leading to endothelial denudation and media smooth muscle cell injury [22]. This arterial wall injury results in direct media SMC exposure to various bioactive molecules, resulting in aggressive SMC migration and proliferation [4, 22]. Further, endothelial cells are the primary barrier between blood and the underlying vessel wall, providing a renewable, non-thrombogenic surface for blood circulation [4, 22, 35]. Without the collective migration of endothelial cells over a site of vessel injury or an implanted stent, the vessel remains thrombogenic and vulnerable to SMC migration and proliferation, contributing thrombotic closure or restenosis [36]. Therefore, it is vital to study and understand the migration of each cell type under injury and non-injury conditions and to examine how individual variables contribute to these processes. The assays in this study provide a valuable tool to further our understanding of how differing conditions, namely injury vs. non-injury, inward vs. outward migration, as well as substrate comparisons, contribute to collective cell migration.

### Study limitations

There are several other factors that influence the migratory behavior of cells. Recent studies suggest that additional conditions such as stress, size and shape of a wound have an impact on the migratory behavior of cells [4, 35, 37, 38]. The shape and size of a wound area contribute to a changing cellular tension from intracellular stress fibers at the wound edge. Studies have shown that with an increasing radius of circular cell area, there are a decreasing number of leader cells. A smaller radius of a wound corresponds to greater curvature and cell tension that increases opportunity for escape of leader cells into the clear zones [36]. However, cell movement is driven by contractile forces generated by the leading edge [39]. Furthermore, for the same reason that greater curvatures produce more leader cells, convex curvature boundaries tend to produce more leader cells than a concave curvature [37].

When analyzing our circular wound area assays, the in-growth assay used a concave boundary with a smaller radius, whereas the out-growth assay used a convex boundary with a larger radius. Our results did not show a consistent increase in migration for the smaller radius, convex boundary assay (in-growth) and therefore concluded that it did not contribute significantly enough to the migration response in comparison to injury and substrate. We did not take the size and shape of the wound areas into account for this study, however, modifications to the assays to create consistent sizes and shapes to more easily tease out these factors can be done in the future.

In this present study, we were unable to tease out the contribution of proliferation to the overall cell growth. Both migration and proliferation have been shown to be positively stimulated by injury and contact de-inhibition as well as contribute to the overall growth of cells [4, 22]. However, the specific contribution of proliferation on migration will be assessed in future studies.

## Conclusion

While migration is a basic process of cellular function, it is dependent upon the integration of multiple factors and individual mechanisms. Injury induces a complex stimulus, resulting in a migration of both endothelial and smooth muscle cells. Migration methods that are able to tease out involved factors are useful for *in vitro* mechanistic studies. Here we utilized three different migration assays to elucidate the contribution of different factors on cell migration, i.e. injury and surface. The cell exclusion assays (in-growth assay and out-growth assay) measure non-injury inward and outward migration, respectively. In contrast, the scrape wound assay measures inward cell migration after cell injury occurs. We hypothesized that the presence of injury and a biologically active surface, gelatin, would yield an increase in cell migration for both SMC and HUVEC. As expected for both cell types, the injury-inducing scrape wound assay provided the highest percent migration at 48 h, followed by the in-growth assay and then the out-growth assay. Additionally, SMCs had higher overall migration than HUVECs for all three assays. We were successfully able to differentiate between wounding and non-wounding, with the difference best demonstrated with the non-injury out-growth assay. Lastly, the presence of a biologically active substrate (gelatin) increased HUVEC migration in all three assays. The gelatin surface provided multiple cell attachment sites that allowed cells to anchor and gain traction for subsequent cell migration. The utilization of these injury and non-injury, as well as inward vs. outward migration assays has allowed us to differentiate the different components of the migratory process (i.e. injury, surfaces) for a variety of cell types (i.e. SMC and HUVEC). Extension of our assay approaches to other cell types may prove useful for controlling variables associated with cell migratory processes and in elucidating the relative contribution of these factors to the cell migration process.

## Methods

### Smooth muscle cell culture

Primary rat SMC cultures were established according to a modification of the method of Ross, et al. [40]. Briefly, rat descending aorta was aseptically harvested, adherent fat and adventitia were removed and aortas were de-endothelialized via passage of an applicator. Aortic tissue was then minced and fragments were incubated (37 °C, 5 % CO_2_) in Dulbecco’s Modified Eagles Medium (DMEM, Life Technologies, Carlsbad, CA) for seven days to allow outgrowth.

Primary SMCs were cultured in T-75 tissue culture flasks (Thermo Scientific, Rochester, NY, USA) with supplemented DMEM. DMEM was supplemented with 10 % fetal calf serum (Life Technologies, Carlsbad, CA, USA), 1 % (*v/v*) antibiotic-antimycotic (Life Technologies, Carlsbad, CA, USA), and 1 % (*v/v*) 0.2 M L-glutamine (Lonza Walkersville, Walkersville, MD, USA). Media was stored at 4 °C for use up to 4 weeks. SMC were grown to 80 % or greater confluency and were passaged with trypsin-versene mixture (Lonza Walkersville, Walkersville, MD, USA) before use in experiments. Only cells between passages 3 and 8 were used.

### HUVEC cell culture

HUVECs were purchased from BD Biosciences (San Jose, CA, USA), and cultured on gelatin-coated T-75 tissue culture flasks with supplemented M199 medium (Life Technologies, Carlsbad, CA, USA). M199 was supplemented with 1 % (*v/v*) 0.2 M L-glutamine, 1.5 % (*v/v*) 1 M HEPES (4-(2-hydroxyethyl)-1-piperazineethanesulfonic acid from Lonza Walkersville, Walkersville, MD, USA), 1.8 % PSG (penicillin-streptomycin-glutamine from Lonza Walkersville, Walkersville, MD, USA), 15 % (*v/v*) fetal calf serum (FBS), sodium bicarbonate (Lonza Walkersville, Walkersville, MD, USA), and heparin salt (Fisher Bioreagents, Fair Lawn, NJ, USA). Endothelial cell growth supplement (Alfa Aesar, Ward Hill, MA, USA) was added to the supplemented M199 to achieve a final concentration of 40 μg/ml. Full media was stored at 4 °C for use up to 4 weeks. HUVECs were grown to 80 % or greater confluency and were passaged using a 50:50 mixture of trypsin-versene and HBSS (Hank’s Balanced Salt Solution from Lonza Walkersville, Walkersville, MD, USA) before being used in experiments. Only cells between passages 3 and 6 were used.

### RVEC cell culture

RVECs were purchased from Cell Biologics, Inc., (Chicago, IL, USA) and were cultured on gelatin-coated tissue culture flasks with the same M199 supplemented media as previously described for HUVEC culture. RVECs were grown to 80 % or greater confluency and were passaged using a 50:50 mixture of trypsin-versene and HBSS (Lonza Walkersville, Walkersville, MD, USA) before being used in experiments. Only cells between passages 3 and 8 were used.

### Substrate surface preparation

Tissue culture treated polystyrene 24-well plates were used for all experiments (Thermo Fisher Scientific, Roskilde, Zealand, DK). In experiments where a gelatin surface was used, 25 μl of dextrose-gelatin-veronal solution (Lonza Walkersville, Walkersville, MD, USA) was coated to cover the entire bottom of each polystyrene well. The solution was dried in a sterile laminar flow hood for 2 h and was kept at 4 °C for a maximum of 1 week. PDMS posts or cylinders are placed above the gelatin-coated well surface in order for the gelatin to remain present beneath both the cell-free and cell confluent areas throughout the assay.

For fluorescent surface preparation, gelatin-FITC was purchased from Thermo Fisher Sci. (Grand Island, NY, USA) and prepared as described above.

### Non-injury Out-growth assay

Pyrex® Cloning Cylinders were purchased from Fisher Scientific (Pittsburgh, PA, USA), with an inner diameter of 4.0 mm, outer diameter of 6.0 mm, and a height of 8.0 mm (Fig. 1a). Cylinders were sterilized via autoclaving and placed under ultraviolet (UV) light for one hour. One cylinder was placed into the center of each well of a 24-well plate prior to cell seeding (Fig. 1c). Cell suspension (50 μl) at a concentration of 100,000 cells/ml was added to the inside of each cylinder. Cells were allowed to seed for 4 h at 37 °C and 5 % CO_2_. Cylinders were then atraumatically lifted out of each well, and 0.5 ml fresh media was added.

Plates were then re-incubated (37 °C and 5 % CO_2_) for desired migration times. After 0, 4, 24 or 48 h of migration, plates were rinsed with 1x phosphate buffered saline (PBS from Fisher Scientific, Fair Lawn, NJ, USA), fixed with Safefix II (Fisher Diagnostics, Middletown, VA, USA), rinsed and stained (Fig. 1d) with 0.1 % toluidine blue (Sigma-Aldrich, St. Louis, MI, USA).

### Non-injury in-growth assay

A 3-D template was designed using Solidworks (Waltham, MA, USA) to create a master of the PDMS (polydimethylsiloxane) form. This mold consisted of two pieces of acrylonitrile butadiene styrene (ABS) that were clamped together and filled with a PDMS mixture (Dow Corning, Phoenix, AZ, USA). After pouring the mold, the pieces were placed in a vacuum chamber for an hour to remove air from the mixture and then placed in 60 °C for at least 2 h to cure. The cured molds were sterilized in 70 % ethanol and then under UV light for 1 h. These molds were made to fit a 24-well plate and were placed in these wells prior to the addition of cells. Cell suspension (0.5 ml) at a concentration of 100,000 cells/ml was added to the wells with the inserts. A sterile 24-well plate filled with de-ionized water (weight of ~120 g) was placed on top of the inserts to secure them in place. The plates were placed in 37 °C and 5 % CO_2_ for 4 h to allow the cells to adhere to the surface. Inserts were then lifted out of the wells, and the plates were placed back in an incubator at 37 °C and 5 % CO_2_ for the desired migration time (0, 4, 24 or 48 h). After migrating, cells were prepared as previously described.

### Injury scrape wound assay

Wooden applicator sticks (1 mm. OD, Baxter, McGaw Park, IL, USA) were sterilized by autoclaving and placed under UV for an hour prior to experiments. Each stick end was examined for flatness and irregularities before use, with non-flat and irregular sticks discarded. Next, 0.5 ml of cells were seeded into each well of a 24-well plate at a concentration of 100,000 cells/ml. Cells were allowed to adhere for 4 h in an incubator at 37 °C and 5 % CO_2_. Media was removed from each well, and the surface was then scraped gently with a sterilized stick end in direct with the plate surface, creating a wound area for cell migration. Wells were gently rinsed with 0.5 ml 1x PBS and replaced with 0.5 ml of supplemented medium. Plates were then re-incubated (37 °C and 5 % CO_2_) for the desired migration time (0, 4, 24 or 48 h). After migrating, cells were prepared as previously described.

Quantification of scrape pressure was calculated by performing the scrape wound assay on a measuring scale to obtain force of scrape and then dividing by the total area of the wooden applicator stick.

### Data analysis

Migration wells were imaged with a Zeiss Axiovert 135 microscope at 4X magnification for quantitative analysis. Each image was taken to include the leading edges of the wound area at each migration time point (0, 4, 24, and 48 h). The extent of migration was calculated by determining the percentage of migration area into the wound or clear zone area after a specified migration period (4, 24, or 48 h) in relation to an initial starting point at 0 h migration. The formula for finding the percent migration when using the non-injury in-migration assay or the injury scrape wound assay is given by:

$$ \begin{array}{l}\%\kern0.5em \mathrm{Migration}=\left[\left({\mathrm{A}}_{\mathrm{initial}}-{\mathrm{A}}_{\mathrm{migration}}\right)/{\mathrm{A}}_{\mathrm{initial}}\right]\times 100\\ {}{\mathrm{A}}_{\mathrm{initial}}=\mathrm{Initial}\kern0.5em \mathrm{clear}\kern0.5em \mathrm{zone}\kern0.5em \mathrm{area}\\ {}{\mathrm{A}}_{\mathrm{migration}}=\mathrm{Clear}\kern0.5em \mathrm{zone}\kern0.5em \mathrm{area}\kern0.5em \mathrm{after}\kern0.5em \mathrm{migration}\end{array} $$

The initial clear zone area (A_initial_) was calculated as the average of the clear zone areas at 0 h migration for each cell type on the substrate surface of interest. Clear zone area (A_migration_) was calculated as an average of the measured areas at each migration time point (4, 24, or 48 h), for each cell type on the substrate surface of interest.

Cell migration studies performed using the non-injury out-migration assays were examined through images captured with a Zeiss Stemi SV11 microscope at 1.2X magnification. The extent of migration was calculated by determining the percentage of outward migration area after a specified migration period (4, 24, or 48 h) in relation to an initial starting point at 0 h migration. The formula for finding the percent migration when using the non-injury out-migration assay is given by:

$$ \begin{array}{l}\%\kern0.5em \mathrm{Migration}=\left[\left({\mathrm{A}}_{\mathrm{migration}}-{\mathrm{A}}_{\mathrm{initial}}\right)/{\mathrm{A}}_{\mathrm{initial}}\right]\times 100\\ {}{\mathrm{A}}_{\mathrm{initial}}=\mathrm{Initial}\kern0.5em \mathrm{cell}\kern0.5em \mathrm{area}\\ {}{\mathrm{A}}_{\mathrm{migration}}=\mathrm{Cell}\kern0.5em \mathrm{area}\kern0.5em \mathrm{after}\kern0.5em \mathrm{migration}\end{array} $$

The initial clear zone area (A_initial_) was calculated as the average of the circular cell areas at 0 h migration for each cell type on the substrate surface of interest. Cell area (A_migration_) was then calculated as an average of the cell areas at each migration time point (4, 24, or 48 h), for each cell type on the substrate surface of interest.

Areas needed for calculating the percent migration were measured by tracing the leading edge boundary of migration with ImageJ software (U.S. National Institutes of Health, Bethesda, Maryland, US). Percentage of cell migration was necessary for comparison between different assays and cell types. Every assay was performed on both gelatin and polystyrene surfaces to produce at least 9 samples at every time point (t = 0, 4, 24, and 48 h). Migration percentages were considered significantly different for *p*-values less than 0.05.

The rate at which the cells migrated was also estimated. This was done by identifying the slope between the averaged percent migration at 24 and 48 h migration time points. The rates are presented as a percentage of migration per hour.

## Additional file

Additional file 1:
**Effect of Cell Density on Migration of Rat Smooth Muscle Cells.** Description of Data: The two graphs show the percent migration of rat smooth muscle cells using the (A) in-growth assay and (B) scrape wound assay. “Normal Density SMC” is used to describe the migration of rat smooth muscle cells at the density used in the present study. “High Density SMC” is used to describe the migration of rat smooth muscle cells when the density was increased to be proportionate to that of the out-growth assay. There was found to be no significant difference (*p* > 0.05) between the normal density and high density SMC migration after 4, 24, or 48 h. Values shown as mean ± standard error. (PDF 58 kb)

## Abbreviations

SMC: Smooth muscle cell
EC: Endothelial cell
HUVEC: Human umbilical vein endothelial cell
PDMS: Polydimethylsiloxane
ECM: Extracellular matrix
FITC: Fluorescein isothiocyanate
RGD: Arginyl glycyl aspartic acid
PDGF: Platelet-derived growth factor
bFGF: Basic fibroblast growth factor
EGF: Epidermal growth factor
TGF-ẞ: Transforming growth factor ẞ-1
IGF-1: Insulin-like growth factor
DMEM: Dulbecco’s Modified Eagle Medium
PBS: Phosphate-buffered solution
ABS: Acrylonitrile butadiene styrene
