# Supplementary material for: Collective cell migration of smooth muscle and endothelial cells: impact of injury versus non-injury stimuli
Source: J Biol Eng. 2015 Oct 15;9:19. doi: 10.1186/s13036-015-0015-y (PMC4606904; doi:10.1186/s13036-015-0015-y)
Supplement: Additional file 1: — Effect of Cell Density on Migration of Rat Smooth Muscle Cells. Description of Data: The two graphs show the percent migration of rat smooth muscle cells using the (A) in-growth assay and (B) scrape wound assay. “Normal Density SMC” is used to describe the migration of rat smooth muscle cells at the density used in the present study. “High Density SMC” is used to describe the migration of rat smooth muscle cells when the density was increased to be proportionate to that of the out-growth assay. There was found to be no significant difference (p > 0.05) between the normal density and high density SMC migration after 4, 24, or 48 h. Values shown as mean ± standard error. (PDF 58 kb) [file 13036_2015_15_MOESM1_ESM.pdf]

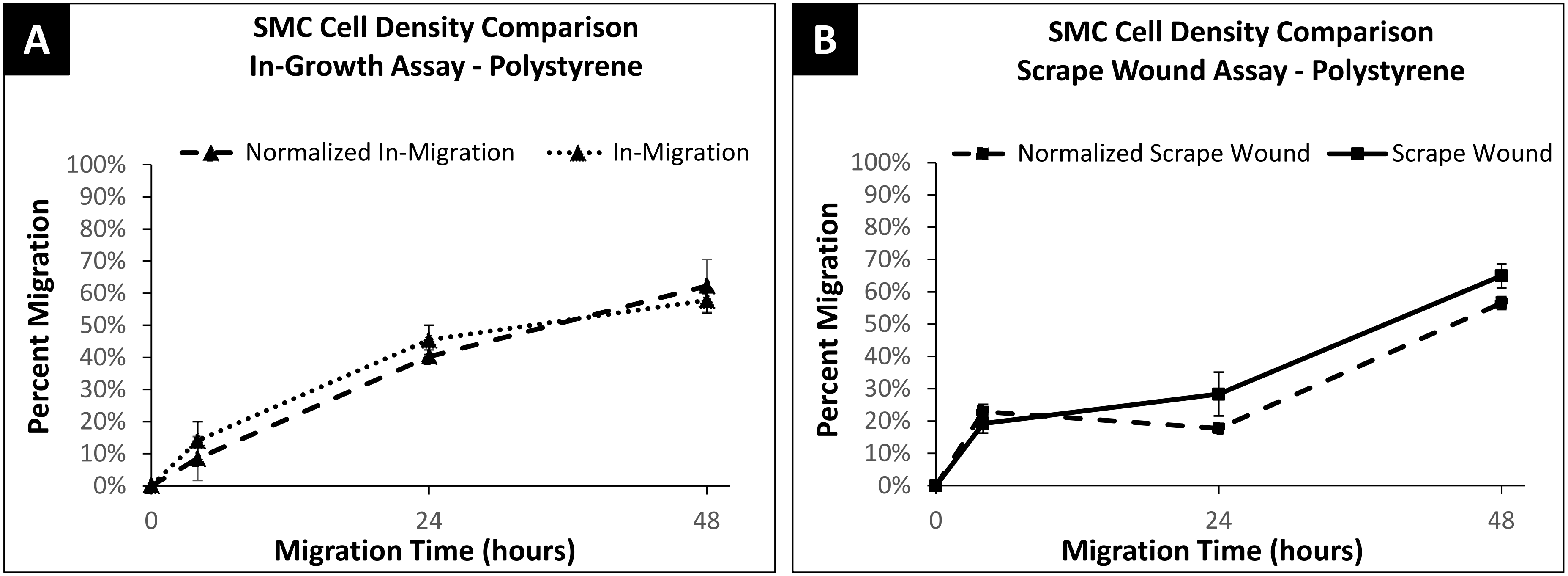
A
SMC Cell Density Comparison
In-Growth Assay - Polystyrene
Normalized In-Migration
In-Migration
Percent Migration
100%
90%
80%
70%
60%
50%
40%
30%
20%
10%
0%
0
24
48
Migration Time (hours)
B
SMC Cell Density Comparison
Scrape Wound Assay - Polystyrene
Normalized Scrape Wound
Scrape Wound
Percent Migration
100%
90%
80%
70%
60%
50%
40%
30%
20%
10%
0%
0
24
48
Migration Time (hours)
